# Behavioral genetics and genomics: Mendel’s peas, mice, and bees

**DOI:** 10.1073/pnas.2122154119

**Published:** 2022-07-18

**Authors:** Hopi E. Hoekstra, Gene E. Robinson

**Affiliations:** ^a^Department of Organismic & Evolutionary Biology, Harvard University, Cambridge, MA 02138;; ^b^Department of Molecular & Cellular Biology, Harvard University, Cambridge, MA 02138;; ^c^Museum of Comparative Zoology, Harvard University, Cambridge, MA 02138;; ^d^HHMI, Harvard University, Cambridge, MA 02138;; ^e^Department of Entomology, University of Illinois at Urbana–Champaign, Urbana, IL 61801;; ^f^Neuroscience Program, University of Illinois at Urbana–Champaign, Urbana, IL 61801;; ^g^Carl R. Woese Institute for Genomic Biology, University of Illinois at Urbana–Champaign, Urbana, IL 61801

**Keywords:** *Apis mellifera*, burrow, division of labor, eugenics, *Peromyscus*

## Abstract

The question of the heritability of behavior has been of long fascination to scientists and the broader public. It is now widely accepted that most behavioral variation has a genetic component, although the degree of genetic influence differs widely across behaviors. Starting with Mendel’s remarkable discovery of “inheritance factors,” it has become increasingly clear that specific genetic variants that influence behavior can be identified. This goal is not without its challenges: Unlike pea morphology, most natural behavioral variation has a complex genetic architecture. However, we can now apply powerful genome-wide approaches to connect variation in DNA to variation in behavior as well as analyses of behaviorally related variation in brain gene expression, which together have provided insights into both the genetic mechanisms underlying behavior and the dynamic relationship between genes and behavior, respectively, in a wide range of species and for a diversity of behaviors. Here, we focus on two systems to illustrate both of these approaches: the genetic basis of burrowing in deer mice and transcriptomic analyses of division of labor in honey bees. Finally, we discuss the troubled relationship between the field of behavioral genetics and eugenics, which reminds us that we must be cautious about how we discuss and contextualize the connections between genes and behavior, especially in humans.

Gregor Mendel’s greatest contribution—now known as Mendel’s laws of inheritance—stemmed from experiments he conducted with garden pea plants (*Pisum sativum*; 1). He chose peas, in part, because they are easy to grow, can be sown each year, and can conveniently be cross-pollinated by hand. In addition, peas have visible polymorphisms: Mendel focused on plants that varied in seed color (green or yellow) and seed morphology (wrinkled or smooth). Each trait had only two distinct forms, which could easily be scored in the offspring of his crosses. By calculating the ratios of each trait’s form across several generations, Mendel could identify consistent patterns, based on dominance and recessivity, of inherited “factors” (now called “genes”). It was the simplicity in the patterns of trait inheritance—single genetic variants that cause simple and specific phenotypic differences—that allowed Mendel to gain key insights into genetic inheritance, laying the foundation for modern-day genetics.

Built on this foundation, we now have a wealth of examples linking genetic variants to, for example, genetic diseases in humans and morphological polymorphisms in a wide diversity of species. Indeed, the genetic lesions for the vast majority of common Mendelian diseases have been identified (2), and we have a rich and growing database of major-effect genes contributing to variants in model organisms (3), domestication traits (4), and adaptations in wild populations (5).

However, it is also clear that not all inheritance patterns follow Mendel’s laws, not all traits have a “simple” genetic basis, and not all are unaffected by environmental conditions. The study of the genetic basis of behavior is faced with all of these challenges—in particular, behavioral variation often (although not always) has complex patterns of inheritance, involving the action and interactions of many genes, and is often strongly influenced by environmental variation. Nonetheless, even Darwin (Chapter 7 in ref. 6) recognized that behavior, like morphological variation, could evolve in the same way—through change over evolutionary time—leading some to suggest that Darwin laid the foundation for behavioral (and hence, behavioral genetic) research for the next century (7).

The first attempt to dissect the genetic basis of behavior was led by Seymour Benzer, who famously used forward-genetic screens to localize chromosomal regions (and ultimately genes) responsible for behavioral differences in mutagenized *Drosophila melanogaster*, for example, the *period* locus that affects circadian rhythm (8; reviewed in ref. 9). Today, approaches that rely on ever more powerful and cheaper genome sequencing technologies enable us to efficiently interrogate all of an organism’s genes and the natural variation therein, which means we can identify DNA variation associated with inherited differences in behavior, as a first step in elucidating the pathway from genotype to phenotype through the nervous system.

New sequencing technologies also opened an additional avenue of study: genome-wide studies of behaviorally related gene expression. Behavioral transcriptomics differs from behavioral genetics in that it does not necessarily focus on inherited differences in behavior. Instead, these studies, especially when followed by functional manipulation, are useful in identifying the neural and/or endocrine mechanisms underlying particular behaviors and understanding how these mechanisms are affected by the environment (10). Thus, the behavioral-transcriptomic approach complements the behavioral genetics approach. Behavioral transcriptomics provides insight into the more dynamic aspects of the relationship between genes and behavior and, together with the more deterministic aspects revealed by discoveries of causal behavior genes, provides a more comprehensive understanding of the relationships between genes and behavior.

To provide an overview of our current understanding of the relationship between genes and behavior, here we review some of the progress that has been made with both approaches: a focus on DNA variation associated with variation in burrowing behavior in deer mice (*Peromyscus* sp.) and studies of brain gene expression and gene regulatory networks (GRNs) as they relate to hormonally regulated division of labor in honey bees (*Apis mellifera*). Readers interested in broader surveys of the field can consult other recent reviews (11–14). Because the study of genes and behavior is a complicated and societally fraught subject, we conclude with a brief discussion of the broader implications of how the legacy of Mendel relates to our current understanding of the relationship between genes and behavior.

## Mendel’s Other Interests: Mice and Bees

**Fig. 1.**
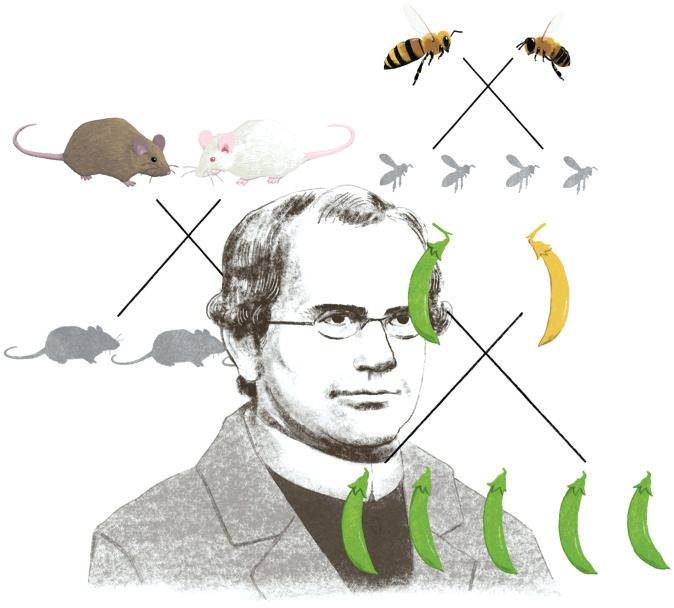
Mendel’s diverse interests. Other organisms, including house mice and honey bees, piqued Mendel’s interest but, for both biological and nonbiological reasons, his ability to perform genetic crosses on those species was limited. Instead, it is from his elegant study of the progeny of pea plants that he derived his laws of inheritance.

In 1843, when Mendel joined a monastery in Brno at the age of 21, he was drawn to both experimentation and the natural world. Specifically, he was interested in understanding how variation—in what we now recognize as simple traits—is inherited from generation to generation to give rise to differences between their offspring (Fig. 1). He initially thought to conduct these experiments in mice, which were popular among mouse fanciers at the time and have a wide range of coat colors that could be easily tracked across generations. In fact, he started to cross wild-type and albino mice in his monastery room (15). However, he never saw the outcome, as once the bishop caught wind of his idea to propagate mice (which, of course, involved sexual reproduction) in the abbey, Mendel’s plans were quickly shut down (15, 16).

In the early 1900s, when Mendel’s laws were first rediscovered (17) and scientists scrambled to confirm his findings in other organisms, Lucien Cuénot, a French biologist, carried out the experiment that Mendel had started nearly a half century earlier. Specifically, Cuénot crossed mice of different coat colors but surprisingly, in one cross, was unable to replicate simple Mendelian inheritance (18). It was later determined that the pigment allele responsible for the color difference between the focal strains was a recessive lethal (19), resulting in a 2:1 ratio of color morphs in offspring derived from a monohybrid cross (rather than an expected Mendelian 3:1 ratio). The pigment allele segregating in what had come to be known as “Cuénot’s odd mice” has since been identified—the *Ay* allele of the *Agouti signaling protein*—and its molecular mechanism revealed (20). Thus, the bishop’s directive likely had a profound effect on the nascent field that would later be known as “genetics,” a term coined by W.B. Bateson, the chief popularizer of Mendel’s ideas following their rediscovery.

Mendel also was an avid beekeeper and tried to study inheritance in honey bees (21). Mendel kept colonies of honey bees at the monastery and was interested in several topics of practical importance, including improving beehive designs and honey production. He also likely performed the first genetic studies of honey bees (22), crossing Cyprian and Carniolan bees to observe effects on a variety of behavioral and physiological traits. It is not exactly clear how he performed the cross, however, because reproduction in honey bees is much more difficult to control than in pea plants. Honey bee queens and drones mate outside in flight; many have tried and failed to coax them to mate in captivity, including Mendel (prompting the development of instrumental insemination of queen bees in the mid-20th century). Moreover, queen honey bees mate with multiple drones, making it impossible for Mendel to have determined the pedigree of the resulting worker bees. Nevertheless, Mendel reported that the first-generation hybrid worker bees were extremely industrious, and the queens were extremely fecund. Given the difficulties, Mendel wisely did not follow up on this early discovery of hybrid vigor, which still today is not well understood at the molecular level, in any species (e.g., 23). However, with the help of sophisticated breeding paradigms, instrumental insemination, and one of the first sequenced insect genomes (24–26), honey bee genetics has made strong advances over the past three decades. Notable discoveries include identifying genomic regions contributing to variation in the propensity to forage for nectar or pollen (27) and the identification and functional validation of the *csd* gene, which underlies haplodiploid sex determination in honey bees (28).

Mendel did not only dodge the complexities of mouse coat color and honey bee reproductive biology, but there are many additional complexities, from a genetic perspective, that can obscure a simple connection between the inheritance of a particular allele and a trait of interest. Such deviations can include peculiarities of specific alleles/genes (e.g., codominance, incomplete dominance), the effect of multiple loci and their interaction (e.g., epistasis, which can lead to incomplete penetrance and/or variable expressivity), or the interaction between an allele and the environment (i.e., environmental effects) or gene-by-environment interactions, to name a few. Most trait variation, including behavior, is anything but simple.

## Behavioral Genetics

The founding of behavior genetics—a field broadly interested in the question of how genetic (and environmental) differences influence behavioral differences—has been attributed largely to Francis Galton, a cousin of Charles Darwin, who in the nineteenth century studied, among other things, the inheritance patterns of “social and intellectual achievement” in a large human pedigree of wealthy British families, concluding there was statistical evidence for a hereditary contribution to differences in achievement among individuals (29). By acknowledging a role for the environment, he also reignited the ongoing “nature versus nurture” debate. The field and Galton’s contributions, however, were later undermined by his leading role in eugenics, a term he coined to describe the idea that selective breeding combined with knowledge about the inheritance of behavior could improve the human species (30, 31). In addition to the deep racial prejudices associated with eugenics, the idea is based on an overly simplistic and erroneous understanding of the inheritance of behavioral variation.

It was not until the mid-20th century that behavioral genetics reemerged as a respectable scientific field. It now is widely accepted in the scientific community that variation in many, if not most, behaviors in animals (including humans) has some genetic influence, although the size of the genetic effect for any particular trait can differ widely. At the time of this rebirth, the genetic contribution to behavioral variation was most often explored either through twin or family studies in humans or in a handful of model animals, which could be studied in the laboratory. In recent years, with the advent of (affordable) genomic approaches, it is increasingly possible to connect genes and their expression to a wide diversity of behaviors in a wide diversity of (even nonmodel) species, allowing for exciting new insights into how behavior evolves in the wild.

## Approaches to Connecting Genetic Variation and Behavioral Variation

One goal of behavioral genetics is to identify the precise genetic contributors to differences in natural behavior. Over the last several decades, behavioral geneticists have been estimating the number and location of genomic regions (those harboring causal mutations) associated with behavioral differences (e.g., 32, 33; reviewed in ref. 34), whereas others focused on the specific roles of known genes, such as neuropeptides, on behavior (e.g., 35, 36). Both approaches—forward genetics, which focuses on determining the genetic basis of a given phenotype, and reverse genetics, which involves genetic manipulations to elucidate gene function by examining changes to phenotypes—have contributed to our understanding of the link between genes and behavior. Now, the field is well poised to combine quantitative genetics and molecular genetics to interrogate the entire genome (using unbiased approaches) to identify specific genes that contribute to complex behavioral variation from a molecular perspective.

The ability to easily score millions of genetic markers or sequence entire genomes across hundreds or thousands of individuals in almost any species (e.g., genotype-by-sequencing approaches) has enabled researchers to more efficiently narrow in on causal genes through a variety of forward-genetic approaches, such as quantitative trait locus (QTL) mapping, genome-wide association studies, or comparisons of genomic differentiation between behaviorally distinct populations (reviewed in ref. 13). For example, using QTL mapping, Ding and colleagues (37) identified an insertion of a retroelement in the regulatory region of an ion channel in the *slowpoke* locus that affects a specific aspect of courtship song (sine song frequency) in *Drosophila*. By generating resequencing data from sweat bees from social and solitary populations, Kocher and colleagues (38) implicate a single genetic variant in the *cis*-regulation of *syntaxin-1*, a gene that mediates synaptic vesicle release, contributing to a social-behavior polymorphism. Or a genome-wide scan of genetic differentiation between two warbler populations pointed to a strong candidate gene, *Vacuolar protein sorting 13A* (*Vps13A*), at which allelic variation may contribute to a binary choice in winter migration path (39). Clearly, new genome-wide genotyping and sequencing approaches have enabled the identification of (candidate) genes, and in some cases mutations, contributing to behavioral variation segregating within and between species, adding to a set of classic examples of the genetics of behavioral polymorphisms [e.g., colony structure in fire ants (40); foraging behavior in *Drosophila* (41)]. These approaches are also applicable to the dissection of even more complex behavioral variation.

## Behavioral Genetics in Deer Mice

While house mice (genus *Mus*), like those that Mendel once considered studying, have long served as a premier model for mammalian behavioral genetics, the study of additional rodent species, which either express more extreme differences in behavior or even behaviors not performed by *Mus*, can serve as a complementary model for the genetic dissection of complex behavioral variation. For example, deer mice (genus *Peromyscus*) have well documented variation in behavior (and other traits), often associated with their local environment (42). This natural behavioral variation combined with the ability to maintain these mice in the laboratory allows for both estimates of the heritability as well as the ability to conduct forward genetics, starting with genetic crosses to document inheritance of behavioral differences. In some cases, large behavioral differences observed in the wild, such as differences in aggression between island and mainland deer mice (43), show no heritable component when offspring of wild mice were reared in a common laboratory environment (44). However, other behaviors have a strong genetic component, such as interspecific differences in parental care: forward-genetic dissection of the parental-care behavior between two *Peromyscus* species, combined with a high-resolution whole-genome sequence and RNA-sequencing data from the hypothalamus, resulted in the localization of a specific gene, *arginine vasopressin* (*Avp*), whose difference in allele-specific expression level leads to differences in parental nest building behavior (45). Still other heritable behavior differences in deer mice have more lessons to share—specifically the evolution of burrowing behavior (46)—thus providing promising opportunities to apply modern molecular genetics to understanding the genetic basis of behavior evolution.

## Genetic Architecture of Burrowing

**Fig. 2.**
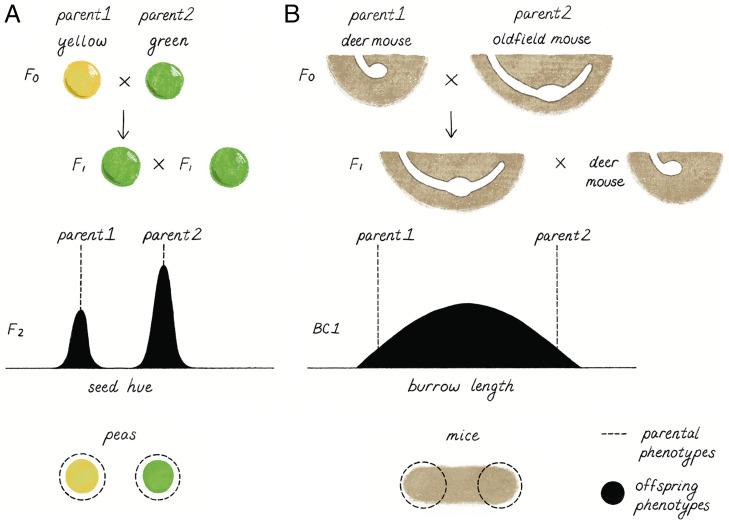
Simple versus complex inheritance. (*A*) Pea seed color, as studied by Mendel, exhibits a simple genetic basis. When pea plants that generate yellow- and green-colored seeds are crossed (F0), the seeds of all progeny are green (F1). When F1 progeny are crossed, the resulting F2 progeny produce either yellow seeds or green seeds, fully recapitulating the F0 parental phenotypes with no intermediates. These results can be explained by a single gene for seed coat color, identified as staygreen (*sgr*; ref. 90), with a dominant green and recessive yellow allele. (*B*) Burrow architecture in *Peromyscus* mice exhibits a more complex genetic basis. When oldfield mice are crossed with deer mice (F0), the resulting offspring dig burrows like oldfield mice (F1). However, when F1 mice are backcrossed with deer mice, BC1 offspring display a continuous distribution of burrow length that spans parental species phenotypes. These results are consistent with multiple, dominant loci from oldfield mice contributing to longer burrows.

Many organisms build structures in their environment (e.g., bird nests, spider webs, beaver dams), which requires a suite of coordinated behaviors. Burrows represent one such architecture, which can provide safety from predators, buffering from environmental fluctuations, and/or a place to store resources (47). All *Peromyscus* use rhythmic head and limb movements to dig their burrows, yet different species consistently produce burrows of a particular size and shape (48, 49). One species in particular, the oldfield mouse (*Peromyscus polionotus*), has an extremely long and stereotyped burrow architecture, which includes an entrance tunnel, nest chamber, and a secondary tunnel, which radiates up toward the ground’s surface but does not penetrate the surface and is used as an escape tunnel (50). It has been hypothesized that this burrow shape may be key to their survival: these mice live almost exclusively in an open habitat (e.g., oldfields, abandoned agricultural fields, and coastal dunes), with little vegetative cover as protection from predators or thermal fluctuations (51). This burrow shape contrasts strikingly with the burrows constructed by other species, which either do not build burrows at all or build small, simple burrows (e.g., the deer mouse, *Peromyscus maniculatus*), with one exception: the Aztec mouse (*Peromyscus aztecus*), which constructs a long burrow (49). Importantly, however, all lack an escape tunnel. Because *Peromyscus* burrowing behavior can be recapitulated in the laboratory in a common soil environment, and some sister-species pairs are interfertile and thus can be intercrossed, this provides an opportunity to determine, as Mendel did with his peas, the inheritance patterns of specific burrow traits across hybrid generations (Fig. 2).

The burrows built by oldfield mice are remarkable, both for their clever design and for the consistency with which they are constructed. Several lines of evidence support a strong genetic component for this particular behavior. First, mice born in the laboratory, despite never witnessing burrow construction or even experiencing soil, produce a species-typical burrow (49, 52). Oldfield mice will even do so when cross-fostered by short burrowing deer mouse parents (53). Even more remarkable, young oldfield mice, starting at 19 d of age (note that pups are weaned at 24 d of age) produce burrows with species-typical shape, only the overall burrow size is smaller; this contrasts with deer mice that do not start building their simple burrows until much later, at 27 d of age (53). In fact, the burrow size and shape constructed by an individual mouse is highly repeatable across trials (49, 54). Both males and females dig burrows, with no differences in burrow architecture between the sexes (55). While it was originally proposed that pregnant females may be most motivated to dig natal burrows (52), there is no evidence for differences in burrow construction between virgin, mated, or pregnant females (56). Burrow length is almost invariant even in the wild, even if burrow depth varies with soil type (i.e., burrows built in heavy loam are shallower than those built in loose sand; ref. 55). Together these data are consistent with the complex burrowing behavior of the oldfield mouse being largely innate.

The burrows constructed by the oldfield mouse and its sister species, the deer mouse (*P. maniculatus*), differ dramatically—both in size and shape (Fig. 2*B*). In the laboratory, oldfield mice produce burrows that are, on average, 40 cm in total length with a 20-cm entrance tunnel; by contrast, deer mice burrows have only a short entrance tunnel of ∼7 cm (49, 55). These two species are interfertile, which offers the opportunity to dissect the genetic basis of these differences in burrowing behavior. Indeed, first-generation (F1) hybrids produce burrows that are statistically indistinguishable from the oldfield mice—long and including an escape tunnel—suggesting the behaviors, and the underlying causal alleles, act in a dominant fashion (Fig. 2*B*; 52, 55). Crossing these F1 hybrids to deer mice produced a generation of backcross (BC1) hybrids, for which 25% of alleles are inherited from oldfield and 75% from deer mice, that resulted in a wide range of burrow architectures, including burrows that matched the parental burrows (i.e., long with an escape tunnel, or short without an escape tunnel) as well as those that differed from the parents (i.e., long without an escape, and short with an escape; Fig. 2*B*). These BC1 burrow phenotypes suggest that, like Mendel’s peas, in which seed color and seed morphology seemed to segregate independently, the major genes affecting burrow length and burrow shape may be unlinked (55). In other words, the complex burrow seems to be subserved by distinct genetic modules.

The genetic architecture of *Peromyscus* burrow variation also showed some clear differences from the simple inheritance patterns that Mendel observed in his peas (Fig. 2). For example, in the BC1 hybrids, burrow length did not have only two discrete states (e.g., long or short), but instead was continuously distributed. This pattern suggested that allelic variation at more than just one gene likely drives the difference in burrow length. Indeed, by genotyping the BC1 population with thousands of markers across the genome, genomic regions were localized in which particular genotypes were predictive of burrow phenotype. There were three regions on three separate chromosomes that correlated with burrow length, and notably a separate fourth chromosome was associated with the presence and/or absence of escape tunnel (55). Together, the three regions accounted for ∼15% of the variation in burrow length. Based on the repeatability of burrowing behavior in the oldfield mice, ∼24% of variation in burrowing can be explained by genetics, suggesting that the three regions account for a majority of the genetic effects; nevertheless, these results also suggest that additional loci and/or environmental effects likely also influence burrowing. Indeed, a recent study intersected alleles that were differentially expressed in the brain both between species and between burrowing and nonburrowing animals with these mapping results to implicate a role for additional small-effect loci that contribute to burrowing difference (57).

These discoveries have been facilitated by high-resolution linkage maps, enabled by efficient and cost-effective genotyping approaches, and, more recently, genome assemblies with gene annotations for these species. Genome assemblies improve the precision of genetic mapping and downstream investigations of the possible causal genes and mutations. Moreover, long-read sequencing now allows us to move beyond surveying only single nucleotide mutations and discover larger structural genetic changes that have been associated with multiple adaptive traits as in *Peromyscus* (58) as well as behavioral polymorphisms [e.g., mating behavior in ruffs (59, 60), reproductive behavior in white-crowned sparrows (61), and social organization in fire ants (62); reviewed in Wellenreuther and Bernatchez (63)].

## Simple versus Complex Genetics

Mendel’s experiments were both elegant and powerful because of their simplicity—the traits were simple and the underlying genetics was simple. A single gene with just two alleles fully explained the inheritance of an easily measured trait (e.g., color of a pea seed) (Fig. 2*A*). Most trait variation, however, is more complex, as is often the case for behavior. Nonetheless, sometimes what is most striking is the contrast between the degree of perceived complexity at the phenotypic versus genotypic level. In the case of burrowing, one can argue that the evolution from a short, simple burrow to a long burrow with intricate engineering (i.e., a well-designed escape hatch) is likely to be complex. Nonetheless, the ability to map a handful of regions in a relatively small mapping population is remarkable, and the possibility of a small number of discrete genetic modules (e.g., relatively large-effect loci that each affect a different aspect of burrow size/shape) suggests a relatively simple genetic architecture. However, this result also does not preclude the contribution of allelic variation at a few or even many small-effect loci to variation in burrowing behavior. Much work remains (and is indeed ongoing) to narrow in on causal genes within these regions, which is arguably the most challenging and time-consuming step of forward-genetic approaches, especially for behavioral traits for which candidate genes may be obscure. In the meantime, it is interesting to imagine what types of genetic changes could lead to a longer burrow—perhaps genetic changes leading to faster-acting or longer-lasting muscle movements, changes in locomotory sequence, or a change in motivation to dig, for example. In any case, whatever the allele(s) may be, it is unlikely that there is a gene or genes “for burrowing.” Most genes are pleiotropic and play multiple roles in organisms, at different timepoints and/or in different tissues. Thus, even for very tractable behaviors, the situation is not as simple as one gene–one trait. But the power of genomics is now letting us investigate the genetic basis of the vast majority of heritable traits, behavior included, that are not as simple as traits in Mendel’s peas.

## Behavioral Genomics

The advent of genomics, now almost 30 y ago, also enabled the development of a new approach to study the connection between genes and behavior, which complements the powerful forward-genetic approaches described earlier. This involves measuring gene expression (most often in the brain) associated with differences in behavior. Behaviorally related gene expression analyses certainly were performed prior to the genomic era, but then they were usually restricted to one or a few genes at a time. Genomics enables entire transcriptomes to be interrogated—from whole brains, brain regions, or individual brain cells—to provide unbiased gene discovery (64, 65). Differences in gene expression associated with differences in behavior result in lists of usually hundreds to thousands of differentially expressed genes (DEGs). Sometimes these DEG lists provide candidate genes for functional studies to go beyond correlation: for example, RNA sequencing of mosquitoes that are attracted to humans versus other mammals pointed to a candidate olfactory receptor later shown with additional experiments to cause difference in host preference (66). However, it is important to note that using transcriptomics alone to find causal genes is often difficult because even a single casual genetic variant can affect the expression of thousands of genes. Nonetheless, annotation tools such as Gene Ontology can be used to provide suggestive insights into the pathways or mechanistic themes that might characterize the differences in behavior. In addition, transcriptomics also can serve as the basis for studies of mechanisms of gene regulation via the modeling of GRNs, an approach discussed in detail later in this work, and can be particularly powerful in understanding the influence of the environment. Thus, without needing to perform crosses, the traditional Mendelian starting point, genomics has dramatically expanded the scope of the study of genes and behavior, enabling many naturally occurring and complex behaviors to be studied (especially those not amenable to laboratory experiments) in many different species at the molecular level.

## Division of Labor in Honey Bee Colonies

The social behaviors of honey bees gave Charles Darwin pause (67). Darwin struggled—for his theory of evolution by natural selection to hold true, he needed to convince himself that what he referred to as the “wonderful instincts” of social insects could evolve through the accumulation of small changes over time (Chapter 7 in ref. 6). Indeed, the inheritance of such complex social behavior of honey bees also fascinated Mendel, as discussed earlier. Both scholars yearned to understand how complex, innate social organization could be inherited over generations, a question we are only now just beginning to unravel from a genetic perspective.

**Fig. 3.**
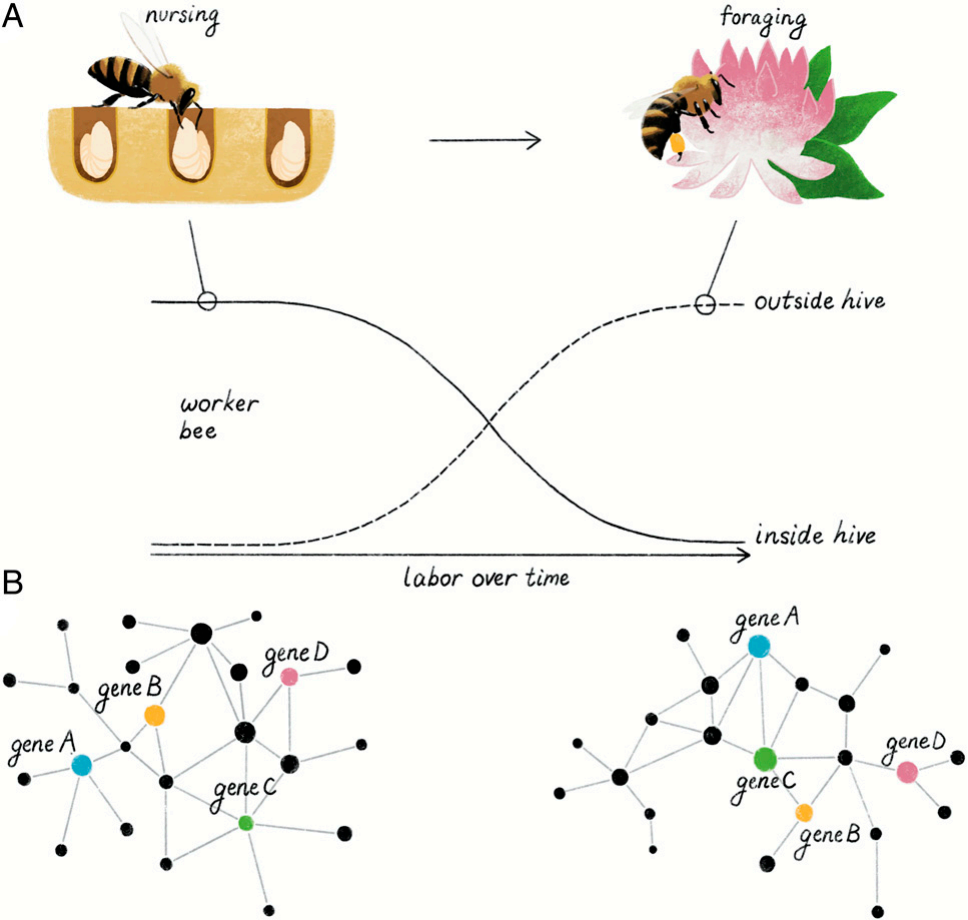
Gene expression networks underlie behavioral plasticity. (*A*) A honey bee worker will begin its adult life working inside the hive as a nurse and then will transition through a middle-age phase of tasks before a shift to working outside the hive as a forager. (*B*) The behavioral changes associated with this labor transition are tied to changes in brain gene expression in thousands of genes. The coordinated changes in brain gene expression result in markedly different gene expression networks between nurse and forager worker bees.

One of the most important components of the intricate organization of the honey bee colony is a division of labor among worker bees, which is based on a process of individual behavioral maturation (Fig. 3*A*; ref. 68). Adult worker honey bees typically live for 4 to 7 wk during the active season; they work in the hive sequentially performing a series of tasks for the first 2 to 3 wk including brood care (“nursing”) and honey processing, and then spend the remainder of their lives defending the hive and searching for nectar and pollen (“foraging”). In addition, honey bee colonies cope with changes in health, age demography, resource availability, and other factors through flexible changes in division of labor based on worker bees accelerating, delaying, or even reversing their behavioral maturation to serve the needs of their colony. This is usually determined by measuring the age at onset of foraging because the transition from working in the hive to foraging outside the hive is particularly sharp.

So far, about a dozen social, nutritional, hormonal, neurochemical, and molecular determinants of age at onset of foraging have been identified, including several genes (69). Each determinant either speeds up or slows down behavioral maturation to cause a precocious or delayed onset of foraging, respectively; the focus here will be on endocrine-related determinants. There also are inherited differences in rate of behavioral maturation (70); while no specific DNA variants have yet been identified, forward-genetic mapping studies, like those described earlier, suggest that the genetic architecture underlying variation in rate of behavioral maturation is highly polygenic (27).

In an early study of division of labor-related brain gene expression (71), and one of the first genome-wide surveys of behaviorally related gene expression in any species, nursing and foraging bees were compared. In addition to their well-known differences in behavior, extensive research has revealed that nurses and foragers differ the most in physiology, brain structure, and brain chemistry relative to all other groups of task specialists in honey bee colonies (72). Given these differences, it was expected that at least some genes will show differences in expression between nurses and foragers. Nonetheless, it was surprising to learn that almost 40% of the roughly 5,500 genes (with the early technology available at the time) with measurable activity in the brain were either up-regulated or down-regulated in nurses compared to foragers. Though only correlative, the differences measured in bees were so robust that it was possible to computationally predict whether a bee was a nurse or forager solely by its brain gene expression profile. Since that report, many studies conducted in a wide range of species report behavioral differences associated with brain expression differences in hundreds or more often thousands of genes.

## Genes Involved in Hormonal Regulation of Division of Labor

Many of the genes differentially expressed in the brain of nurses and foragers are related to hormonal regulation of division of labor (73). Division of labor among worker honey bees has evolved by co-opting elements of the basic insect reproductive system, especially juvenile hormone (JH) and vitellogenin (Vg) (74). In most insect species, an increase in the blood levels of JH is associated with the onset of reproduction, but in honey bees it is associated with the onset of foraging. Hormone treatments that mimic this increase cause precocious foraging, while removal of the JH-producing corpora allata delays it. In addition, Vg inhibits JH production, JH inhibits Vg production, and the two of them together form a double repressor feedback loop to control age at onset of foraging (75).

So far, four JH-related genes have been identified that differ in expression between nurses and foragers and also cause changes in behavioral maturation (however, no specific DNA variants in behaviorally related genes have yet been identified in honey bees). The four are *vg*, *ultraspiracle* (*usp*), *broad*, and *fushi tarazu transcription factor* 1 (*ftz-f1*). The *vg* gene encodes Vg, a lipoprotein that is one of the most abundant yolk proteins in insect eggs. In honey bees, Amdam, Page, and colleagues (27) have demonstrated that *vg* has evolved novel roles related to immune function, longevity, nutrition, and behavior; knockdown of vg in the abdomen by RNA interference (RNAi) increases blood levels of JH and leads to precocious foraging (75, 76). *Usp* is involved in orchestrating the transcriptional response to JH; it also is part of a gene family known to encode proteins regulating metabolism and nutritional physiology in many species. This gene was of particular interest because changes in nutritional physiology are linked to honey bee behavioral maturation; for example, starvation is a factor that induces precocious foraging. Moreover, *usp* expression is up-regulated in the fat body following JH treatment, and knockdown of *usp* by RNAi causes a delay in the onset of foraging (77). *Broad* and *ftz-f1* also have been linked to JH transcriptional effects: *broad* encodes a protein that integrates signals from diverse endocrine pathways including JH and Vg, while the expression of *ftz-f1* itself is regulated by *broad*. Knockdowns of the expression of *broad or ftz-f1* in the brain by RNAi also cause delays in the onset of foraging (78). These four genes represent only a small fraction of the molecular machinery underlying hormonal regulation of division of labor, but studies about their regulation already have yielded important insights into the dynamic relationship between genes and behavior.

## GRNs and Division of Labor

Genes and neurons work together to make brain function possible, and both function within networks. GRNs are located inside each cell in the brain (and other tissues) and coordinate gene expression. Neuronal networks (NNs) coordinate the activity of neural circuits that transmit electrochemical signals from one neuron to another. We are only just beginning to understand how GRNs and NNs integrate brain activity to control behavior (79).

It is assumed that the honey bee genome contains a comparable number of transcription factors (TFs) to *Drosophila*, roughly 700. How many TFs regulate the expression of each gene, and how many genes are regulated by each TF? There is not yet enough information for firm numbers, but it is thought that each gene is regulated by about 5 to 100 TFs (80), and each TF regulates the expression of tens to hundreds of genes, sometimes including other TFs. It is thus very clear that gene regulation occurs in hierarchical, cascading, networks, with tiers of TFs working in combination to regulate the expression of their target genes.

*Usp*, *broad*, and *ftz-f1* code for TFs, and as expected, each of these TFs regulates many genes (Fig. 3*B*; refs. 69 and 81). *vg* RNAi abdomen knockdown results in changes in the expression of thousands of genes, in both the fat body and brain (76). Vg, however, is not a TF; these effects are thus indirect, due to interactions between Vg and associated TFs. A computational analysis of the genomes of 10 different species varying in level of social complexity also has implicated *usp* and *broad* in the evolution of social life in bees (82).

Computational analyses enable genome-scale analyses to find the location of all copies of DNA binding motifs associated with each TF to identify the genes predicted to be regulated by each TF. This approach has yielded two important insights thus far. First, regulation of gene expression by TFs involves complex and variable combinatorial rules. Recall that honey bee behavioral maturation is affected by a variety of social, nutritional, hormonal, neurochemical, and molecular determinants, only some of which were discussed earlier. A study of 11 of these determinants showed that they collectively influence the expression of overlapping sets of genes in the brain (73). Using newly created bioinformatic tools, Ament and colleagues (77) showed that these overlapping genes are in turn regulated by overlapping sets of TFs. But the precise combination of TFs varied for the different determinants, and the ways that TFs are involved appear to follow different forms of computational logic (e.g., “and, “or,” and “not” gates). For example, two *cis*-regulatory motifs together predict gene expression responses to the 11 determinants of behavioral maturation but follow different Boolean combinatorial rules. These findings hint at a highly complex regulatory code that governs how the same genes regulate different behaviors (Fig. 3*B*).

Second, regulatory relationships between TFs and target genes are context dependent. Based on a meta-analysis of brain gene expression profiles from 853 individual adult worker honey bees exhibiting 48 distinct behavioral states associated with division of labor, the manner in which a TF and its target genes are coexpressed in the brain is predicted to vary with behavioral state (e.g., whether the measurements were made from a bee engaged in nursing or foraging; ref. 80). Focusing on *broad* and *ftz-f1*, Hamilton and colleagues (78) found results consistent with this prediction: patterns of coexpression between *broad* and *ftz-f1* and their respective targets indeed varied with behavioral state, and also with experimentally induced changes in *broad* and *ftz-f1* brain expression due to RNAi. For example, a brain GRN predicts a positive correlation between the expression of *broad* and the target gene *GB15608* for bees in an aggressive behavioral state associated with hive defense, but a negative correlation for bees engaged in foraging (78). Changes in regulatory relationships between TFs and target genes have already been discovered to occur at evolutionary time scales (83). These new results now extend this concept to shorter time scales associated with neurobiology and behavior.

## Behavioral Genetics and Genomics

**Fig. 4.**
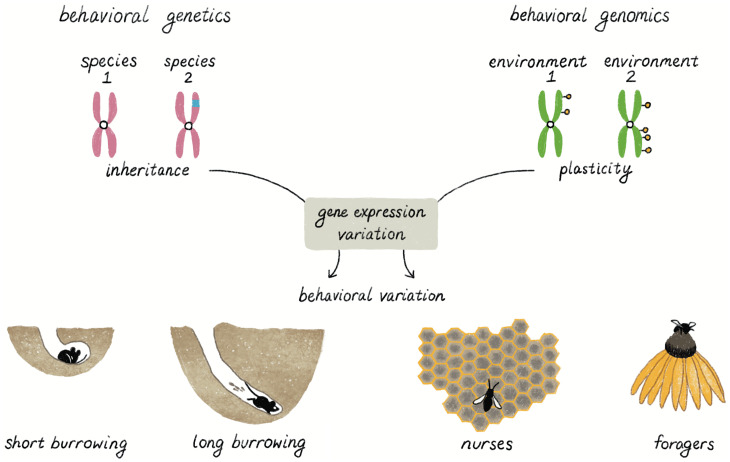
Behavioral genetics and behavioral genomics as complementary approaches. Both behavioral genetics and behavioral genomics investigate the link between genes and behavioral variation. Behavioral genetics, as exemplified by *Peromyscus* burrowing, seeks to connect inherited genetic variation to behavioral variation. Changes in genetic sequence are represented by different colors of chromosomes (pink vs. green). Behavioral genomics, as exemplified by honey bee division of labor, seeks to connect changes in gene expression to behavioral plasticity. Behavioral plasticity can be caused by changes in the environment (external or internal), which can lead to, for example, epigenetic modifications (yellow circles) without changing DNA sequence (purple). Both changes in DNA sequence and the environment result in changes in gene regulation, and, ultimately, behavior.

A complete understanding of the relationship between genes and behavior requires probing the consequences of genetic variation at multiple levels: from DNA sequences to brain gene expression, to brain connectivity, to neuronal firing dynamics, to behavioral variation. Behavioral genetics provides information on causal genes and alleles, whereas behavioral genomics can help identify mechanistic roles of these alleles through transcriptomic and GRN analyses; both approaches are necessary to gain a complete picture of how genes contribute to behavior (Fig. 4). While there are a growing number of examples in which allelic variation at genes is associated with behavior, there are many fewer cases in which we also have a clear understanding of how those genes act in or on neural circuits to cause differences in behavior, especially in the context of evolutionary changes. We do know that experience-dependent changes in brain gene expression can help prepare individuals to adjust their behavior to new environmental conditions (72, 84). The use of transcriptomics to discover evolutionarily conserved “genetic toolkits” for behavior is yet another approach to facilitate a comprehensive understanding of the relationship between genes and behavior (85).

## Reconsidering the Relationship between Genes and Behavior

Mendel’s pioneering studies provided the foundation for the science of genetics. His choice of traits with simple inheritance eventually led others to adopt a “genes for” shorthand for an expectation of a direct, specific, and causal relationship between a gene and a trait. For behavior, it is important to acknowledge that this perspective has been all too easily misused to promote inequities, racism, and genocide (86). The studies reviewed here highlight that the relationship between genes and behavior is more complicated than suggested by the “genes for” shorthand. To be sure, there are indeed excellent examples of individual genes that have DNA variants with specific and causal relationships to a behavior, some of which affect the expression of many other genes (e.g., 35, 45, 66, 87, 88; reviewed in ref. 13). But the studies described in depth earlier serve as examples of how “genes for” thinking is misguided. There are no genes that specify behavior; rather, genetic variation acts to change biochemical and cellular pathways that alter neuronal circuits to result in behavioral variation and, ultimately, behavioral evolution. Because most genes are pleiotropic, changes in any one gene often affect many traits. Moreover, even the most fundamental elements of gene regulation are not fixed, and we have as yet little knowledge of the dynamic and context-dependent codes that govern the operation of behaviorally related GRNs. In addition, human genome-wide association studies reveal that associations between specific alleles and behavioral variation are heavily dependent on the populations that are studied (i.e., the genetic background in which alleles are found), providing another example of “context-dependent” connections between genes and behavior (89). Given that behaviors are the product of both nature and nurture, it is clear that both inherited and environmental influences affect the precise and ever-changing relationships between genes and behavior (Fig. 4).

Understanding the complex relationship between genes and behavior—and how these connections may vary among individuals, populations, and species—is a grand challenge for both science and society. Studies such as those reviewed here are pushing scientists to move past the outdated “genes for” paradigm, and it is critically important for scientists to work hard to enable the public to recognize and understand the underlying science. In this way, we can honor the legacy of Mendel’s pioneering discoveries, highlight the scientific and societal progress we have made since, and identify the important work that still must be done.

## Data Availability

There are no data underlying this work.

## References

[1] G. Mendel, Versuche über Pflanzen-Hybriden (Experiments in plant hybridization). Verhandlungen des naturforschenden Vereines in Brünn (Abhandlungen) 4, 3–47 (1866).

[2] McKusick-Nathans Institute of Genetic Medicine, Johns Hopkins University, OMIM Online Mendelian Inheritance in Man: An online catalog of human genes and genetic disorders. https://www.omim.org. Accessed 15 January 2022.

[3] U. Irion, C. Nüsslein-Volhard, Developmental genetics with model organisms. Proc. Natl. Acad. Sci. U.S.A. 119, e2122148119 (2022). 10.1073/pnas.2122148119 PMC9335277 35858396

[4] L. Andersson, M. Purugganan, Molecular genetic variation of animals and plants under domestication. Proc. Natl. Acad. Sci. U.S.A. 119, e2122150119 (2022). 10.1073/pnas.2122150119 PMC9335317 35858409

[5] K. Bomblies, C. L. Peichel, Genetics of adaptation. Proc. Natl. Acad. Sci. U.S.A. 119, e2122152119 (2022). 35858399 10.1073/pnas.2122152119 PMC9335183

[6] C. Darwin, On the Origin of Species by Means of Natural Selection, or the Preservation of Favoured Races in the Struggle for Life (John Murray, London, ed. 1, 1859). PMC5184128 30164232

[7] B. Thierry, Darwin as a student of behavior. C. R. Biol. 333, 188–196 (2010). 20338536 10.1016/j.crvi.2009.12.007

[8] R. J. Konopka, S. Benzer, Clock mutants of *Drosophila melanogaster*. Proc. Natl. Acad. Sci. U.S.A. 68, 2112–2116 (1971). 5002428 10.1073/pnas.68.9.2112 PMC389363

[9] J. Weiner, Time, Love, Memory: A Great Biologist and His Quest for the Origins of Behavior (Vintage Books, New York, 2000).

[10] National Academies of Sciences, Engineering, and Medicine, Next Steps for Functional Genomics: Proceedings of a Workshop (National Academies Press, Washington, DC, 2020). 33284567

[11] I. Anreiter, H. M. Sokolowski, M. B. Sokolowski, Gene-environment interplay and individual differences in behavior. Mind Brain Educ. 12, 200–211 (2017).

[12] T. A. McDiarmid, A. J. Yu, C. H. Rankin, Beyond the response-High throughput behavioral analyses to link genome to phenome in *Caenorhabditis elegans*. Genes Brain Behav. 17, e12437 (2018). 29124896 10.1111/gbb.12437

[13] N. Niepoth, A. Bendesky, How natural genetic variation shapes behavior. Annu. Rev. Genomics Hum. Genet. 21, 437–463 (2020). 32283949 10.1146/annurev-genom-111219-080427

[14] N. Jourjine, H. E. Hoekstra, Expanding evolutionary neuroscience: Insights from comparing variation in behavior. Neuron 109, 1084–1099 (2021). 33609484 10.1016/j.neuron.2021.02.002

[15] H. Iltis, Life of Mendel (Allen and Unwin, 1932), pp. 92, 105.

[16] R. M. Henig, The Monk in the Garden: The Lost and Found Genius of Gregor Mendel, the Father of Genetics (Houghton Mifflin Harcourt, 2000).

[17] A. Berry, J. Browne, Mendel and Darwin. Proc. Natl. Acad. Sci. U.S.A. 119, e2122144119 (2022). 35858395 10.1073/pnas.2122144119 PMC9335214

[18] L. Cuénot, Les races pures et leurs combinaisons chez les souris. Arch. Zool. Exp. Gén. 4, cxxiii–cxxxii (1905).

[19] W. E. Castle, C. C. Little, On a modified Mendelian ratio among *yellow* mice. Science 32, 868–870 (1910). 17830668 10.1126/science.32.833.868

[20] E. J. Michaud, S. J. Bultman, L. J. Stubbs, R. P. Woychik, The embryonic lethality of homozygous lethal yellow mice (*Ay/Ay*) is associated with the disruption of a novel RNA-binding protein. Genes Dev. 7, 1203–1213 (1993). 8319910 10.1101/gad.7.7a.1203

[21] N. L. Carreck, Gregor Mendel the beekeeper. Am. Bee J. 159, 167–169 (2019).

[22] W. E. Kerr, H. H. Laidlaw, Advances in Genetics, M. Demerec, Ed. (Academic Press, NY, 1947), vol. VIII.

[23] J. Liu, M. Li, Q. Zhang, X. Wei, X. Huang, Exploring the molecular basis of heterosis for plant breeding. J. Integr. Plant Biol. 62, 287–298 (2020). 30916464 10.1111/jipb.12804

[24] Honeybee Genome Sequencing Consortium, Insights into social insects from the genome of the honeybee *Apis mellifera*. Nature 443, 931–949 (2006). 17073008 10.1038/nature05260 PMC2048586

[25] C. G. Elsik ; HGSC production teams; Honey Bee Genome Sequencing Consortium, Finding the missing honey bee genes: Lessons learned from a genome upgrade. BMC Genomics 15, 86 (2014). 24479613 10.1186/1471-2164-15-86 PMC4028053

[26] A. Wallberg , A hybrid de novo genome assembly of the honeybee, *Apis mellifera,* with chromosome-length scaffolds. BMC Genomics 20, 275 (2019). 30961563 10.1186/s12864-019-5642-0 PMC6454739

[27] R. E. Page Jr., O. Rueppell, G. V. Amdam, Genetics of reproduction and regulation of honeybee (*Apis mellifera* L.) social behavior. Annu. Rev. Genet. 46, 97–119 (2012). 22934646 10.1146/annurev-genet-110711-155610 PMC4159717

[28] M. Beye, M. Hasselmann, M. K. Fondrk, R. E. Page, S. W. Omholt, The gene *csd* is the primary signal for sexual development in the honeybee and encodes an SR-type protein. Cell 114, 419–429 (2003). 12941271 10.1016/s0092-8674(03)00606-8

[29] F. C. Galton, Hereditary Genius: An Inquiry into Its Laws and Consequences (MacMillan and Co., London, 1869).

[30] F. C. Galton, Human Faculty (Macmillan and Co., London, 1883).

[31] F. Galton, Eugenics: Its definition, scope, and aims. Am. J. Sociol. 10, 1–25 (1904).

[32] J. M. Wehner, R. A. Radcliffe, B. J. Bowers, Quantitative genetics and mouse behavior. Annu. Rev. Neurosci. 24, 845–867 (2001). 11520920 10.1146/annurev.neuro.24.1.845

[33] J. Flint, Analysis of quantitative trait loci that influence animal behavior. J. Neurobiol. 54, 46–77 (2003). 12486698 10.1002/neu.10161

[34] R. A. York, Assessing the genetic landscape of animal behavior. Genetics 209, 223–232 (2018). 29563148 10.1534/genetics.118.300712 PMC5937184

[35] L. J. Young, Frank A. Beach Award. Oxytocin and vasopressin receptors and species-typical social behaviors. Horm. Behav. 36, 212–221 (1999). 10603285 10.1006/hbeh.1999.1548

[36] C. E. Barrett , Variation in vasopressin receptor (*Avpr1a*) expression creates diversity in behaviors related to monogamy in prairie voles. Horm. Behav. 63, 518–526 (2013). 23370363 10.1016/j.yhbeh.2013.01.005 PMC3602142

[37] Y. Ding, A. Berrocal, T. Morita, K. D. Longden, D. L. Stern, Natural courtship song variation caused by an intronic retroelement in an ion channel gene. Nature 536, 329–332 (2016). 27509856 10.1038/nature19093

[38] S. D. Kocher , The genetic basis of a social polymorphism in halictid bees. Nat. Commun. 9, 4338 (2018). 30337532 10.1038/s41467-018-06824-8 PMC6194137

[39] D. P. L. Toews, S. A. Taylor, H. M. Streby, G. R. Kramer, I. J. Lovette, Selection on *VPS13A* linked to migration in a songbird. Proc. Natl. Acad. Sci. U.S.A. 116, 18272–18274 (2019). 31451666 10.1073/pnas.1909186116 PMC6744891

[40] K. G. Ross, L. Keller, Genetic control of social organization in an ant. Proc. Natl. Acad. Sci. U.S.A. 95, 14232–14237 (1998). 9826683 10.1073/pnas.95.24.14232 PMC24356

[41] M. B. Sokolowski, Foraging strategies of *Drosophila melanogaster*: A chromosomal analysis. Behav. Genet. 10, 291–302 (1980). 6783027 10.1007/BF01067774

[42] N. L. Bedford, H. E. Hoekstra, *Peromyscus* mice as a model for studying natural variation. eLife 4, e06813 (2015). 10.7554/eLife.06813 PMC4470249 26083802

[43] Z. T. Halpin, T. M. Sullivan, Social interactions in island and mainland populations of the deer mouse, *Peromyscus maniculatus*. J. Mammal. 59, 395–401 (1978).

[44] F. Baier, H. E. Hoekstra, The genetics of morphological and behavioural island traits in deer mice. Proc. Biol. Sci. 286, 20191697 (2019). 31662081 10.1098/rspb.2019.1697 PMC6842855

[45] A. Bendesky , The genetic basis of parental care evolution in monogamous mice. Nature 544, 434–439 (2017). 28424518 10.1038/nature22074 PMC5600873

[46] C. K. Hu, H. E. Hoekstra, *Peromyscus* burrowing: A model system for behavioral evolution. Semin. Cell Dev. Biol. 61, 107–114 (2017). 27496333 10.1016/j.semcdb.2016.08.001

[47] M. H. Hansell, The ecological impact of animal nests and burrows. Funct. Ecol. 7, 5–12 (1993).

[48] JN Layne, LM Ehrhart. Digging behavior of four species of deer mice (*Peromyscus*). American Museum Novitates No. 2429 (American Museum of Natural History, New York, 1970).

[49] J. N. Weber, H. E. Hoekstra, The evolution of burrowing behavior in deer mice. Anim. Behav. 77, 603–609 (2009).

[50] F. B. Sumner, J. J. Karol, Notes on the burrowing habits of *Peromyscus polionotus*. J. Mammal. 10, 213–215 (1929).

[51] R. J. Esher, J. L. Wolfe, The effects of temperature and housing on water balance in a burrowing mouse, *Peromyscus polionotus*. J. Comp. Physiol. 133, 241–245 (1979).

[52] W. D. Dawson, C. E. Lake, S. S. Schumpert, Inheritance of burrow building in *Peromyscus*. Behav. Genet. 18, 371–382 (1988). 3219114 10.1007/BF01260937

[53] H. C. Metz, N. L. Bedford, Y. L. Pan, H. E. Hoekstra, Evolution and genetics of precocious burrowing behavior in *Peromyscus* mice. Curr. Biol. 27, 3837–3845.e3 (2017). 29199077 10.1016/j.cub.2017.10.061

[54] N. L. Bedford, “The genetics of burrowing behavior in *Peromyscus* mice in the lab and the field,” Doctoral dissertation, Harvard University, Boston (2019).

[55] J. N. Weber, B. K. Peterson, H. E. Hoekstra, Discrete genetic modules are responsible for the evolution of complex burrowing behaviour in deer mice. Nature 493, 4202–4405 (2013). 10.1038/nature11816 23325221

[56] N. L. Bedford , Behavioural mechanisms underlying the evolution of cooperative burrowing in *Peromyscus* mice. bioRxiv [Preprint] (2019). 10.1101/731984 (Accessed 25 June 2022).

[57] C. K. Hu , *cis*-Regulatory changes in locomotor genes are associated with the evolution of burrowing behavior. Cell Rep. 38, 110360 (2022). 35172153 10.1016/j.celrep.2022.110360

[58] E. R. Hager , A chromosomal inversion drives evolution of multiple traits in deer mice. bioRxiv [Preprint] (2021). 10.1101/2021.01.21.427490 (Accessed 25 June 2022).

[59] S. Lamichhaney , Structural genomic changes underlie alternative reproductive strategies in the ruff (*Philomachus pugnax*). Nat. Genet. 48, 84–88 (2016). 26569123 10.1038/ng.3430

[60] C. Küpper , A supergene determines highly divergent male reproductive morphs in the ruff. Nat. Genet. 48, 79–83 (2016). 26569125 10.1038/ng.3443 PMC5218575

[61] E. M. Tuttle , Divergence and functional degradation of a sex chromosome-like supergene. Curr. Biol. 26, 344–350 (2016). 26804558 10.1016/j.cub.2015.11.069 PMC4747794

[62] J. Wang , A Y-like social chromosome causes alternative colony organization in fire ants. Nature 493, 664–668 (2013). 23334415 10.1038/nature11832

[63] M. Wellenreuther, L. Bernatchez, Eco-evolutionary genomics of chromosomal inversions. Trends Ecol. Evol. 33, 427–440 (2018). 29731154 10.1016/j.tree.2018.04.002

[64] G. E. Robinson, C. M. Grozinger, C. W. Whitfield, Sociogenomics: Social life in molecular terms. Nat. Rev. Genet. 6, 257–270 (2005). 15761469 10.1038/nrg1575

[65] G. E. Robinson, R. D. Fernald, D. F. Clayton, Genes and social behavior. Science 322, 896–900 (2008). 18988841 10.1126/science.1159277 PMC3052688

[66] C. S. McBride , Evolution of mosquito preference for humans linked to an odorant receptor. Nature 515, 222–227 (2014). 25391959 10.1038/nature13964 PMC4286346

[67] F. R. Prete, The conundrum of the honey bees: One impediment to the publication of Darwin’s theory. J. Hist. Biol. 23, 271–290 (1990).

[68] G. E. Robinson, Regulation of division of labor in insect societies. Annu. Rev. Entomol. 37, 637–665 (1992). 1539941 10.1146/annurev.en.37.010192.003225

[69] S. A. Ament , New meta-analysis tools reveal common transcriptional regulatory basis for multiple determinants of behavior. Proc. Natl. Acad. Sci. U.S.A. 109, E1801–E1810 (2012a). 22691501 10.1073/pnas.1205283109 PMC3387076

[70] N. W. Calderone, R. E. Page, Genotypic variability in age polyethism and task specialization in the honey bee, *Apis mellifera* (Hymenoptera: Apidae). Behav. Ecol. Sociobiol. 22, 17–25 (1988).

[71] C. W. Whitfield, A.-M. Cziko, G. E. Robinson, Gene expression profiles in the brain predict behavior in individual honey bees. Science 302, 296–299 (2003). 14551438 10.1126/science.1086807

[72] A. Zayed, G. E. Robinson, Understanding the relationship between brain gene expression and social behavior: Lessons from the honey bee. Annu. Rev. Genet. 46, 591–615 (2012). 22994354 10.1146/annurev-genet-110711-155517

[73] C. W. Whitfield , Genomic dissection of behavioral maturation in the honey bee. Proc. Natl. Acad. Sci. U.S.A. 103, 16068–16075 (2006). 17065327 10.1073/pnas.0606909103 PMC1622924

[74] A. R. Hamilton, H. Shpigler, G. Bloch, D. E. Wheeler, G. E. Robinson, “Endocrine influences on the organization of insect societies” in Hormones, Brain, and Behavior, D. Pfaff, M. Joels, Eds. (Academic Press, New York, ed. 3, 2017), pp. 421–451.

[75] C. M. Nelson, K. E. Ihle, M. K. Fondrk, R. E. Page, G. V. Amdam, The gene vitellogenin has multiple coordinating effects on social organization. PLoS Biol. 5, e62 (2007). 17341131 10.1371/journal.pbio.0050062 PMC1808115

[76] M. M. Wheeler, S. A. Ament, S. L. Rodriguez-Zas, G. E. Robinson, Brain gene expression changes elicited by peripheral *vitellogenin* knockdown in the honey bee. Insect Mol. Biol. 22, 562–573 (2013). 23889463 10.1111/imb.12043

[77] S. A. Ament , The transcription factor *ultraspiracle* influences honey bee social behavior and behavior-related gene expression. PLoS Genet. 8, e1002596 (2012b). 22479195 10.1371/journal.pgen.1002596 PMC3315457

[78] A. R. Hamilton , Social behavior is associated with transcriptional regulatory plasticity in the brain. J. Exp. Biol. 222, jeb200196 (2019). 31138635 10.1242/jeb.200196 PMC6679348

[79] S. Sinha , Behavior-related gene regulatory networks: A new level of organization in the brain. Proc. Natl. Acad. Sci. U.S.A. 117, 23270–23279 (2020). 32661177 10.1073/pnas.1921625117 PMC7519311

[80] D. Marbach , Predictive regulatory models in *Drosophila melanogaster* by integrative inference of transcriptional networks. Genome Res. 22, 1334–1349 (2012). 22456606 10.1101/gr.127191.111 PMC3396374

[81] S. Chandrasekaran , Behavior-specific changes in transcriptional modules lead to distinct and predictable neurogenomic states. Proc. Natl. Acad. Sci. U.S.A. 108, 18020–18025 (2011). 21960440 10.1073/pnas.1114093108 PMC3207651

[82] K. M. Kapheim , Social evolution. Genomic signatures of evolutionary transitions from solitary to group living. Science 348, 1139–1143 (2015). 25977371 10.1126/science.aaa4788 PMC5471836

[83] D. S. Lorberbaum , An ancient yet flexible *cis*-regulatory architecture allows localized Hedgehog tuning by patched/Ptch1. eLife 5, e13550 (2016). 27146892 10.7554/eLife.13550 PMC4887206

[84] I. M. Traniello, G. E. Robinson, Neural and molecular mechanisms of biological embedding of social interactions. Annu. Rev. Neurosci. 44, 109–128 (2021). 34236891 10.1146/annurev-neuro-092820-012959

[85] M. C. Saul , Cross-species systems analysis of evolutionary toolkits of neurogenomic response to social challenge. Genes Brain Behav. 18, e12502 (2019). 29968347 10.1111/gbb.12502 PMC6314924

[86] C. Zimmer, She Has Her Mother’s Laugh: The Powers, Perversions, and Potential of Heredity (Dutton, New York, 2018).

[87] M. M. Lim , Enhanced partner preference in a promiscuous species by manipulating the expression of a single gene. Nature 429, 754–757 (2004). 15201909 10.1038/nature02539

[88] C. F. Kent, T. Daskalchuk, L. Cook, M. B. Sokolowski, R. J. Greenspan, The *Drosophila foraging* gene mediates adult plasticity and gene-environment interactions in behaviour, metabolites, and gene expression in response to food deprivation. PLoS Genet. 5, e1000609 (2009). 19696884 10.1371/journal.pgen.1000609 PMC2720453

[89] J. J. Lee ; 23andMe Research Team; COGENT (Cognitive Genomics Consortium); Social Science Genetic Association Consortium, Gene discovery and polygenic prediction from a genome-wide association study of educational attainment in 1.1 million individuals. Nat. Genet. 50, 1112–1121 (2018). 30038396 10.1038/s41588-018-0147-3 PMC6393768

[90] Y. Sato, R. Morita, M. Nishimura, H. Yamaguchi, M. Kusaba, Mendel’s green cotyledon gene encodes a positive regulator of the chlorophyll-degrading pathway. Proc. Natl. Acad. Sci. U.S.A. 104, 14169–14174 (2007). 17709752 10.1073/pnas.0705521104 PMC1955798

